# Antioxidant-Rich Natural Raw Materials in the Prevention and Treatment of Selected Oral Cavity and Periodontal Diseases

**DOI:** 10.3390/antiox10111848

**Published:** 2021-11-21

**Authors:** Jolanta Pytko-Polończyk, Magdalena Stawarz-Janeczek, Agata Kryczyk-Poprawa, Bożena Muszyńska

**Affiliations:** 1 Department of Integrated Dentistry, Faculty of Medicine, Institute of Dentistry, Jagiellonian University Medical College, Montelupich Street 4, 31-155 Kraków, Poland; jolanta.pytko-polonczyk@uj.edu.pl; 2 Department of Inorganic and Analytical Chemistry, Faculty of Pharmacy, Jagiellonian University Medical College, Medyczna Street 9, 30-688 Kraków, Poland; 3 Department of Pharmaceutical Botany, Faculty of Pharmacy, Jagiellonian University Medical College, Medyczna Street 9, 30-688 Kraków, Poland; muchon@poczta.fm

**Keywords:** antioxidant-rich natural raw materials, natural products, dentistry, periodontal diseases, dental caries

## Abstract

Antioxidant-rich natural raw materials have been used for thousands of years in traditional medicine. In the past decade, there has been increasing interest in naturotherapy, which is a practice of using products with a natural origin. Natural products can be effective in the treatment and prevention of oral and dental diseases, among others. Such raw materials used in dentistry are characterized by antioxidant, anti-inflammatory, antibacterial, antiviral, antiedematous, astringent, anticoagulant, dehydrating, vitaminizing, and—above all—regenerative properties. Reports have shown that a relationship exists between oral diseases and the qualitative and quantitative composition of the microbiota colonizing the oral cavity. This review aimed to analyze the studies focusing on the microbiome colonizing the oral cavity in the context of using natural raw materials especially herbs, plant extracts, and isolated biologically active compounds as agents in the prevention and treatment of oral and periodontal diseases such as dental caries as well as mucosal changes associated with salivary secretion disorder. The present work discusses selected plant ingredients exhibiting an antioxidant activity with potential for the treatment of selected oral cavity and periodontal diseases.

## 1. Introduction

Since the late 20th century, microorganisms (microbiome) residing in the oral cavity and their impact on human health have been extensively studied by researchers [1]. It has been demonstrated that the oral microbiome represents the total genome of all microorganisms found in the body. Unfortunately, the oral microbiome includes not only commensal and symbiotic microorganisms but also pathogenic ones [2,3]. It should be emphasized that there is a need for research analyzing the influence of the oral microbiome, among others, on the functioning of the human body [4].

In the physiological state, the microbial homeostasis in the oral cavity is preserved by two mechanisms: local and systemic. The local mechanism ensures the continuity of the mucosa, proper exfoliation of the oral epithelium, and optimal saliva secretion whereas the systemic mechanism regulates the cellular immunity [5]. Disruption of the microbial homeostasis and thus the ecology of the ecosystem inhabiting the oral cavity has been shown to cause dysbiosis of beneficial microorganisms [6,7,8], resulting in the development of diseases [4,7,8,9] such as periodontitis, dental caries [10], and fungal infections [4,7,8,9]. Moreover, homeostatic disturbances may increase the risk of developing systemic comorbidities or worsen the course of the existing ones [4,7]. These comorbidities include Alzheimer’s disease, cardiovascular disease, esophageal and colorectal cancer, rheumatoid arthritis, pancreatic cancer, diabetes, cystic fibrosis, bacterial endocarditis, aspiration pneumonia and osteomyelitis in children, a preterm low birth weight or premature birth, strokes, and oxidative stress [4,11,12].

Studies on oral microbiota have shown that approximately 700 different microbial species are present in the oral cavity [13,14]. However, only 57% of these bacterial species have been identified and described [11] in the human microbiome database (Human Oral Microbiome Database (HOMD)). The HOMD contains 15 types of bacterial domains [4]. About 96% of the taxa constitute six major phyla: *Firmicutes*, *Bacteroidetes*, *Proteobacteria*, *Actinobacteria*, *Fusobacteria* [15,16], and *Spirochaetes*. The microorganisms that are most commonly found in the oral cavity are *Streptococcus* [17]. Utter et al. (2020) proved the systemic occurrence of different species of bacteria that belong to the same genus, and are thus genomically different, in the oral cavity. This suggests that different sections of the oral cavity are colonized by different specific but related bacteria [18]. Several oral bacteria have also been recently identified. These include *Scardovia wiggsiae* (a causative agent of dental caries), *Streptococcus gordonii*, and *Filifactor alocis* (both are causative agents of periodontitis) [8]. In the case of patients who were diagnosed with periodontitis in the oral cavity, higher levels of *Porphyromonas gingivalis, Treponema denticola,* and *Tannerella forsythia* (the so-called red complex) were observed [13]. *Porphyromonas gingivalis* is a Gram(–) anaerobic bacterium. It is able to modify the immune response of the host. Its major virulence mediator (for example, gingipains) participates in host interactions. It is able to enter the vasculature through an ulcerated epithelium [19]. Periopathogens are capable of causing disease in conditions of dysbiosis (the hypothesis of microbial shift) [13].

Scientific studies have confirmed that in addition to bacteria, several species of fungi, mycoplasmas, animal protists (formerly protozoa), and viruses are present in the oral cavity at a physiological equilibrium [5,13]. Animal protists found in the oral cavity include *Entamoeba gingivalis* and *Trichomonas tenax* [4]. Fungal species colonizing the oral cavity are of the genus *Candida*, mainly *Candida albicans*, which is found in 75% of the human population [16]. Furthermore, yeasts belonging to different genera such as *Cladosporium*, *Aspergillus*, *Fusarium*, *Cryptococcus*, *Filobasidiella*, and *Aureobasidium* have been detected in the oral cavity [4,11]. The presence of a few archaebacteria, mainly methanogenic ones (methanogens) [11] including *Methanobrevibacter oralis*, *Methanobacterium curvum/congolense*, and *Methanosarcina mazeii* [4], has also been reported.

Numerous works have indicated that the microbiome composition varies depending on the location in the oral cavity [12] as well as the health status of individuals [1,11], which is determined by comorbidities [19], nutrition and habits [11], and lifestyle [20]. Other important factors influencing the microbiome composition are oral health status [12], age [13], physical activity, and the innate immunity of the patients as well as environmental determinants. Differences in oral conditions including temperature, salivary flow rate, and pH [20] as well as the oxygen and nutrient availability must also be considered in the analysis of the microbiome composition [11]. The composition of microorganisms in the oral cavity can change during daily life activities such as eating, breathing (including air conditioning), and kissing [20]. Saliva plays an important role in maintaining the microbial homeostasis and a favorable pH for microorganisms. It also provides the microorganisms with nutrients whilst acting as a source of receptors necessary for the bacteria to adhere to the oral surface as well as a source of antimicrobial cystatins, lactoferrin (a protein inhibiting microbial growth), and immune components. In addition, due to its acid-buffering properties, it facilitates tooth remineralization. However, the composition and amount of saliva secreted are affected by periodontal disease and also depend on medications such as antibiotics. Several bacteria in saliva and plaque have been shown to carry antibiotic and antiseptic resistance genes [21].

The application of medicinal plants has been mainly associated with therapeutic practices and dental hygiene for thousands of years in traditional medicine [22,23]. Over the past decade, there has been a significant increase in interest in naturotherapy, which involves the use of naturally derived products as a viable and safe alternative to antibiotics and synthetic products in the prevention and treatment of oral and periodontal diseases. This is due to the fact that natural raw materials exhibit antimicrobial activity against a variety of microorganisms including carious and periodontal pathogens. Moreover, as bacterial species have developed a resistance to dental antibiotics over the years, there is a need to utilize natural compounds for preventing the growth, adhesion, and colonization of bacteria in the oral cavity. Oxidative stress plays an important role in the etiology and course of numerous diseases that can cause oral cavity problems. One way to prevent homeostasis disorders that occur as a result of an excessive production of pro-oxidative substances is to include ingredients with antioxidant properties during therapy. Several compounds such as phenolic derivatives and flavonoids as well as terpenes, catechin tannins, unsaturated fatty acids, vitamins, and bio-elements exhibit an antioxidant activity. The present work discusses selected plant ingredients exhibiting an antioxidant activity with potential for use in the treatment of selected oral cavity and periodontal diseases.

## 2. Antioxidant-Rich Natural Raw Materials in the Treatment and Prevention of Oral Cavity Diseases

Due to their ability to prevent plaque formation, herbs, plant extracts, and biologically active substances isolated from them have been used for the prevention and treatment of oral and periodontal diseases. Several studies on bacterial species known to be involved in the etiology of oral and dental diseases have tested and confirmed the antimicrobial activity of natural substances. Many of these substances have potential applications in dentistry. These include cinnamon bark oil, clove bud oil, papier mâché extracts, and selected components of these extracts such as cinnamaldehyde and eugenol. Extracts and compounds isolated from *Salvadora persica*, *Juglans regia*, *Vaccinium macrocarpon*, *Camellia sinensis*, *Morus alba*, *Trachyspermum ammi*, *Piper betle*, *Vitis vinifera*, *Azadirachta indica*, *Sanguinaria canadensis*, *Myristica fragrans*, *Pistacia vera*, *Artocarpus lakoocha*, *Polygonum cuspidatum*, *Helichrysum litoreum*, *Rosmarinus officinalis*, *Mentha spicata*, *Eugenia caryophyllata*, and *Eucalyptus globulus*, among others, have been reported to have a biofilm-inhibiting activity [24]. Extracts of plants such as *Drosera peltata*, *Abies canadensis*, *Albizia julibrissin*, *Chelidonium majus*, *Ginkgo biloba*, *Juniperus virginiana*, *Pinus virginiana*, *R. officinalis*, *Sassafras albidum*, *Coptidis rhizoma*, *Hamamelis virginiana*, *Tanacetum vulgare*, *Breynia nivosus*, *Allium sativum*, *Harungana madagascariensis*, and *Thuja plicata* have been demonstrated to inhibit the development of oral pathogens thus limiting plaque formation and reducing bacterial adhesion as well as alleviating the symptoms of oral diseases [25]. Natural compounds act on oral infections by inhibiting the bacterial growth (bacteriostatic effect), reducing the number of bacteria (bactericidal effect), preventing microbial adhesion, inhibiting water-soluble glucan production, inhibiting amylases, and disrupting the biofilm [25].

Taking into account the high prevalence of oral diseases, an increased bacterial resistance to antibiotics and chemotherapeutics (including penicillins, cephalosporins, erythromycin, tetracycline, and metronidazole) [26], and the adverse effects of certain antimicrobial drugs that are currently used in dentistry, there is a need to search for alternative safe, effective, and economically beneficial methods for the prevention and treatment of oral and periodontal diseases. This review summarizes the effects of different natural products that have the ability to inhibit the growth of pathogens in the oral cavity, affect microbial adhesion to the oral surface, limit the development of biofilms and plaque, and reduce the symptoms of oral and periodontal diseases such as dental caries and periodontitis as well as counteracting mucosal infections and an impaired salivary secretion.

### 2.1. Dental Caries and Periodontal Disease

According to the data of the World Health Organization (WHO), oral diseases affect as many as 3.5 billion people worldwide [27]. The most prevalent oral infection is decay [28], which is especially observed in permanent teeth, but the decay of milk teeth has also been diagnosed in more than 530 million children [27]. Studies indicate that the second most common oral condition is periodontal disease [28], which affects as much as 10% of the world’s population [27]. It has been shown that tooth decay and periodontal disease often coexist.

In a workshop organized by the European Federation of Periodontology (EFP) and the European Organization for Caries Research (ORCA) in Spain in late 2016, the participants highlighted the importance of dental caries and periodontal disease and announced the Perio-Caries project. This project aimed to disseminate knowledge about the role of microorganisms in the development of caries and periodontal disease as well as methods to prevent these conditions [8].

The WHO defines dental caries as a pathological process that leads to the decalcification and proteolytic breakdown of the hard tissues of the tooth resulting in cavity formation [29,30,31]. Caries develops due to various causes including an increase in the number of acid-forming bacteria. Periodontitis, on the other hand, is caused by proteolytic and anaerobic bacteria.

Despite many common features, caries and periodontitis differ in their mechanism of formation. Their development is influenced not only by the biofilm but also by individual and environmental factors [8]. Periodontitis is distinguished from caries by its chronic, multifactorial inflammatory nature [32].

Raw materials of natural origin are used to treat dental caries as well as gingivitis and periodontitis. The natural products commonly used by physicians for the treatment of caries and its complications including endodontitis are eugenol, zinc oxide, gutta-percha, mastic [33,34], myrrh, rosin, and thymol—a component of temporary fillings (dentin). 

Plant materials containing essential oils are widely used in dentistry. Essential oils are volatile substances that can change into a gaseous form at an ambient temperature. They mainly contain mono-, sesqui-, and diterpenoids, phenolics, and other volatile compounds. Essential oils can be found in all organs of oil plants (leaves, flowers, fruits, rhizomes, and bark). They are obtained by steam distillation or by pressing and sometimes are additionally rectified. The oils obtained from plants are characterized by disinfectant, bactericidal, viricidal, antifungal, anti-inflammatory, analgesic, and astringent properties. The most important natural raw materials containing essential oils applied in dentistry are *Syzygium aromaticum* Thunb. (formerly *E. caryophyllata*), *Salvia officinalis* L., *Matricaria chamomilla* L., *Mentha piperita* L., *Thymus serpyllum* L., *Eukaliptus* (*Eucalyptus L’Hér.*), *Melissa officinalis* L., *Foeniculum vulgare* Mill, and *Cinnamomum cassia* (L.). 

The main biologically active components of clove oil obtained from *S. aromaticum* Thunb include eugenol (about 95%), benzoic acid, and terpenes (pinene and limonene). Eugenol is used in medicine as it exhibits disinfectant, bactericidal, viricidal, antifungal, anti-inflammatory, analgesic, and astringent activities. It can effectively treat deep caries and indirect pulp capping when applied as a paste mixed with zinc oxide. In endodontics, eugenol is used as a component of the paste used for the treatment of pulp disorders [33]. It is also used to precipitate insoluble silver salts during the impregnation of dentin with silver nitrate [33,34]. Eugenol acts by inhibiting inflammatory neurotransmitters such as prostaglandins and leukotrienes and, through its ability to diffuse through dentin, it exhibits analgesic, anesthetic, and anti-inflammatory effects. 

In the case of sage (*S. officinalis* L.), the raw material used in dentistry is the leaves, which contain about 3% of a volatile oil comprising thujone, cineole, camphor, and pinene, among others. Sage is also rich in tannins (that inhibit Gram(+) bacteria), flavonoids, and organic acids and, hence, exhibits an anti-inflammatory effect by reducing the permeability of the blood capillaries [33,35,36]. 

The inflorescence (anthodium) of common chamomile (*M. chamomilla* L.) yields an essential oil with anti-inflammatory, anti-allergenic, disinfectant, and analgesic properties. The main active constituents of chamomile oil are chamazulene, α-bisabolol, and spiroether [37]. 

The raw material obtained from peppermint (*M. piperita* L.) is the leaves. The main ingredient in the leaves is peppermint oil [33,38,39,40], which contains menthol and ascorbic acid (25 mg%), carotene (up to 40 mg%), rutin (14 mg%), apigenin, betaine, and oleanolic and ursolic acid. Menthol is a monoterpenoid with strong anesthetic, bactericidal, odor-freshening, and anti-inflammatory properties. By acting on the cold receptors, it provides a cooling sensation to the inflamed tissue [33,39,40]. 

Thyme (*T. serpyllum* L.) is characterized by antibacterial and dehydrating properties. The primary constituent of its oil is the phenolic compound thymol, which exhibits an antibacterial activity. In dentistry, preparations containing thyme or thyme oil are used for rinsing the mouth and throat to treat inflammations [41,42]. Thymol is also used as a component in dentin (temporary dental fillings) [33].

The leaves of eucalyptus (*E. L’Hér*.) are used as a raw material for obtaining eucalyptus oil, which is used in the food, pharmaceutical, and cosmetic industries. The plant shows antiseptic and antibacterial properties against Gram(–) and (+) bacteria. Its oil is widely used in oral hygiene products (antiseptics, toothpastes, and chewing gums) due to its strong but pleasant fragrance and disinfectant properties [43,44]. 

The leaves of lemon balm (*M. officinalis* L.) are the source of melissa oil, which contains citral, a substance that gives the herb a lemon scent and bitter taste. Due to its antibacterial and antiviral properties, melissa oil is used in the production of toothpastes [45]. 

Chinese cinnamon (*C. cassia* (L.) J. Presl) is a source of cinnamon oil. Due to its astringent, antiseptic, and odor-eliminating properties, cinnamon oil is recommended for mouth rinsing to treat gum and throat disorders and inflamed tonsils.

Lemon oil extracted from *Citrus limon* (L.) Burm exhibits antimicrobial properties against bacteria, fungi, and certain viruses [46]. 

The roots of elecampane (*Inula helenium* L) contain about 3.5% of essential oil, inulin, phytosterols, and mineral salts. Both *I. helenium* and *F. vulgare* Mill (fennel) have antiseptic properties and are thus used for mouth rinsing after tooth extractions as well as to treat gum inflammations [33,47]. 

Bee products, especially propolis and honey, have gained an increasing interest from doctors and researchers all over the world. Among the modern natural methods of treatment, apitherapy is very popular. Propolis is widely used for the prevention and treatment of oral diseases. Its antibacterial activity has been confirmed in in vivo and in vitro studies against *Streptococcus mutans* and *Streptococcus sobrinus*, which is based on the inhibition of the glycosyltransferase activity [48]. Propolis has also been shown to be effective in healing surgical wounds, preventing caries, treating dentin hypersensitivity and aphthous ulcers, and as an ingredient in root canal and mouthwash solutions. However, it is not recommended for people allergic to pollen or bee stings, or those suffering from asthma. The composition of propolis is determined by its geographical origin, among others, and the safety of its application should be confirmed by further studies [49].

Due to its antimicrobial properties, the effect of the bulb extract of *A. sativum* L. (garlic) has been tested against oral cavity-colonizing bacteria. It was found that garlic extract (containing 220 μg/mL of allicin) showed an inhibitory effect on the tested strains of Gram(–) bacteria (garlic: minimum inhibitory concentration (MIC): 36–1.1 mg/mL; allicin: mean MIC: 4.1 μg/mL) whereas it was less effective against Gram(+) bacteria (garlic: MIC: 143–36 mg/mL; allicin: mean MIC: 27.5 μg/mL). Based on an analysis of the time curves of *S. mutans* and *Porphyromonas gingivalis*, it was found that the bactericidal activity began immediately in the case of *P. gingivalis* whereas for *S. mutans* it occurred with a delay of several hours. Garlic extract also inhibited the trypsin-like and total protease activities of *P. gingivalis* by 93% and 95%, respectively. Moreover, it is known to inhibit the growth of pathogens in the oral cavity and may, therefore, have therapeutic applications, especially for periodontitis [50]. An *A. sativum* bulb showed a high antibacterial activity against *Staphylococcus aureus* and *Streptococcus mutans*. The MIC of crude garlic bulbs varied widely and this clearly showed that the bacteria exhibited a different level of susceptibility to secondary metabolites. The MIC value ranged between 20 ± 2 mg/mL and 120 ± 6 mg/mL. An *A. sativum* bulb can be effectively used to treat periodontal and dental caries infections [51]. The use of garlic to treat oral candidiasis and recurrent aphthous ulcers has also shown success without the complications associated with a traditional therapy [52]. Mohammad et al. conducted an examination of the clinical and radiographic effects of *A. sativum* oil and formocresol in a vital pulpotomy in the primary teeth. The comparison showed that there was no significant difference in the clinical and radiographic success rates of vital pulpotomies in primary molars treated with either *A. sativum* oil or formocresol. *A. sativum* oil offered a good healing potential and left the remaining pulp tissue healthy and functioning. A vital pulpotomy with *A. sativum* oil had a 90% success rate whereas one with formocresol was 85% [53].

Xylitol is a sugar alcohol naturally found in plants and used as an artificial sweetener in many food products. Its anticaries properties were tested against *S. mutans*, *Streptococcus saliviarius*, and *Streptococcus sanguis*. It was found that xylitol inhibited the growth of *S. mutans* but had no effect on the other streptococci that are part of the oral cavity flora [54].

Zinc oxide, another substance of natural origin, exhibits an antiseptic effect and has been used in pediatric dentistry, among others, for pulpotomies and the pulpectomy of deciduous teeth and indirect pulp capping. It is also a component of the temporary material dentin [33] and is used as a sealant for filling root canals [55].

Toothpastes, chewing gums, and rinses are used in the prevention of dental caries and oral diseases [13]. In toothpastes, various natural substances such as aloe vera [56], propolis, extracts from the above-mentioned oil plants, fennel, common lemon [13], mastic (a natural resin extracted from the balsam of the *Pistacia lentiscus* tree), and myrrh are used. Similar to the preparation of chewing gums, natural products including gutta-percha, eucalyptus oil [33], and propolis [57] are utilized. Oral refreshing rinses include mastic and other natural active ingredients such as menthol, methyl salicylate, eucalyptol, and thymol as antimicrobial and antiseptic agents [33]. To eliminate bad breath, an infusion of *Urtica dioica* is used for mouth rinsing as the herb has a deodorizing effect due to its high chlorophyll content.

Mastic [33], myrrh [38,58], tannins (sage leaf, oak bark, tormentil rhizome, and raspberry leaf), thymol, elecampane, fennel, and Chinese cinnamon have been used in the treatment of gingivitis and periodontitis, among others [33].

*Porphyromonas gingivalis* is a major pathogen causing periodontitis and can even lead to tooth loss. An evaluation of the antimicrobial activity of 109 extracts obtained from 21 plant species used for oral hygiene and exhibiting an antimicrobial activity against *P. gingivalis* by Carrol showed that 21 extracts from 11 plants caused more than a 90% inhibition against *P. gingivalis* [59]. These extracts were prepared from the following raw materials: *P. lentiscus*, *Zanthoxylum armatum*, *Vicia faba*, *Carya alba*, *J. regia*, *Citrus sinensis*, *Morella cerifera*, and *S. albidum* [59].

It is now known that honey exhibits a significant bactericidal activity against many oral pathogens that are biofilm components. Studies have indicated that honey exhibits a bacteriostatic activity against *S.* *mutans*, *Actinomyces viscosus*, *S. sobrinus*, *Fusobacterium nucleatum*, and *P. gingivalis*. A study reported that manuka honey from New Zealand showed a significant bactericidal activity against *S. mutans*. It was also observed that manuka honey reduced the growth of *S. mutans* on saliva-coated hydroxyapatite disks and glass surfaces. These findings indicate the anticaries and antimicrobial potential of honey [60].

Another natural raw material that can be used to treat periodontal disease is *Curcuma xanthorrhiza*. It was shown that xanthorrhizol isolated from the methanolic extract of turmeric roots displayed the highest antibacterial activity against caries-causing *Streptococcus* as well as against *A. viscosus* and *P. gingivalis*, which are the causative agents responsible for periodontitis. On the other hand, *C. albicans* and *Lactobacillus* were found to be resistant to the antibacterial effect of xanthorrhizol. However, based on the results obtained, it could be concluded that xanthorrhizol can be used as a component in dental products to prevent oral diseases [61].

Extracts from the edible fungus *Lentinus edodes* (shiitake) and from *Cichorium intybus*, Italian red chicory, were found to exhibit a bacteriostatic and a weakly bactericidal activity, respectively. These natural compounds may, therefore, be useful for improving daily oral hygiene [62].

### 2.2. Salivary Secretion Disorder

Saliva plays an important role in maintaining microbial homeostasis. Salivary secretion disorder or a saliva deficiency may lead to a shift in the microbial balance toward pathogenic microorganisms such as *C. albicans* or *S. mutans* [5]. The affected patients mainly complain of dryness in the mouth (xerostomia) [5,63] and may also experience burning and swelling of the salivary glands. Moreover, salivary secretion disorder increases the susceptibility to caries and the risk of developing lip angle inflammation or exfoliative lip inflammation [5]. This condition can be treated by diagnosing and treating the underlying disease and providing a symptomatic treatment to stimulate salivary secretion. Generally, sugarless chewing gums and lemon-, mint-, and cinnamon-flavored candies as well as saliva substitutes, vitamin E oil, and citric acid solutions are recommended [5,63]. In a few patients, an increased saliva secretion is found. In such cases, rinsing the oral cavity with a tannin solution can be effective [64].

Studies have analyzed the use of natural raw materials to increase salivary secretion and improve comfort in patients with a dry mouth (xerostomia). The clinical efficacy of lycopene-enriched virgin olive oil in a spray form was tested in elderly patients with drug-induced xerostomia (*n* = 60). The results showed that its topical application significantly improved the xerostomia symptoms in patients without affecting the salivary flow rate [65]. A clinical study conducted on 20 patients with type II diabetes mellitus, with an aim of evaluating the effect of a herbal ginger spray on reducing a dry mouth, showed that the application of a ginger spray caused a significant increase in the mean saliva flow rate (*p* < 0.001) [66].

Herbal rinses can be effective in alleviating mouth burning. The management of a burning mouth remains a challenge as knowledge of the etiology and pathophysiology is still limited. Capsaicin, a naturally derived substance, causes a significant reduction in pain levels when administered systemically. It was found that the topical application of this compound or its use as a rinse for 1 week resulted in effective pain relief although 32% of patients reported a severe stomach pain [67]. The use of capsaicin is also associated with other possible side effects including tissue necrosis [68].

## 3. Prevention of Oral Diseases

Oral conditions can be prevented by the mechanical removal of the biofilm. This is due to the fact that pro-health behaviors such as physical activity and proper nutrition [8] as well as preventing addictions to substances affecting oral health such as nicotine [13] play a major role. It is important to implement inflammation prophylaxis in addition to regular check-ups combined with the mechanical removal of plaque as well as iatrogenic factors (e.g., overhanging fillings and improper prosthetic restorations). It is necessary to teach patients about proper oral hygiene and motivate them to adapt oral hygiene activities. The patients should also be taught how to avoid addictions, choose an appropriate brushing method and additional tools (such as dental floss, single-ended or interdental brushes, or irrigators), and introduce prophylactic or therapeutic mouthwashes into the daily oral hygiene routine according to their individual needs. In addition, dietary and nutritional advice should be given as the nutritional status influences not only the course of an infection but also the functioning of the immune system. Through food, the body can be provided with beneficial antioxidants and vitamins. Deficiencies of vitamins such as A, C, E, coenzyme Q10, and bioflavonoids as well as folic acid, fatty acids (abundant in fish), and minerals such as copper and selenium can cause periodontal changes. A vitamin A and folic acid deficiency sensitizes the gingival pockets to inflammatory factors. For example, green tea has an inhibitory effect on *P. gingivalis* and reduces gingivitis as well as eliminating odors from the mouth. A diet rich in carbohydrates and fats promotes a faster accumulation of tartar. Furthermore, it is important to include more hard foods in the diet as the consumption of mushy foods is associated with poorer hygiene [69]. Vitamin D can influence the way in which the ignition is switched due to its anti-inflammatory and anti-systemic effects [70]. In the studies described by Pandav et al. in 2021, the authors suggest that coenzyme Q10 might be able to prevent chronic periodontitis. However, they stress the need for further research to help establish the role of coenzyme Q10 in preventing periodontal disease [71]. Cagetti et al. reviewed the systematic and meta-analysis of the links between vitamin intake and periodontitis. Due to the lack of sufficient evidence, however, it was not possible to make recommendations on consumptions [72].

Studies over the past few years have shown that certain foods contain a number of ingredients with antimicrobial and antiplatelet effects [73]. Phenolic compounds are the major group of substances exhibiting these effects. A diet rich in polyphenols may help to prevent changes in the oral bacterial flora and thus the development of oral conditions, especially caries and gingivitis [73,74]. The antibacterial activity of aqueous propanone (P70) extracts from plants containing polyphenols was tested against *S. mutans*. The lowest MIC values were determined for red grape, green tea, and sloe berry extracts. It was also found that the tested extracts reduced the adhesion of *S. mutans* to glass [75]. The regular consumption of certain foods can facilitate the modification of the oral microbial community toward a less periodontopathogenic microbiota. These foods include honey/propolis, green tea, aloe vera, cranberries, coffee, cocoa, grapes, olive oil, berries, or licorice [76,77,78]. Studies analyzing the effects of polyphenols on the prevention of oral conditions such as dental caries, gingivitis, periodontal disease, candidiasis, oral sores, oral mucositis, oral lichen planus, leukoplakia, and oral cancer have indicated that there is a lack of randomized clinical trials focusing on the protective role of polyphenols in oral diseases. Preclinical studies have demonstrated the beneficial effects of polyphenols in the treatment of most common oral diseases (caries, periodontitis, and candidiasis) [74].

Oxidative stress could be predisposed to inflammatory processes, which can be counteracted by bioactive compounds present in natural raw materials [79]. The occurrence of diseases in the oral cavity is influenced by a change in the composition of the bacterial flora, which is also related to substances contained in the saliva [5]. Risk factors may influence the development of periodontitis including nicotinism, obesity, the host genotype, and poor oral hygiene [13]. Therefore, the main goal in the prevention and treatment of oral diseases should be to improve general immunity [5,80] and health [5]. In dentistry, natural raw materials are mostly used in the form of rinses, tinctures, infusions, toothpastes, drops, and gels. Among the active compounds, essential oils, monoterpenoids, phenolic compounds, gums, resins, tannins, flavonoids, anthocyanins, carotenoids, chlorophyll, vitamins, and elements (especially zinc) have been distinguished so far [33]. For many years, patients have been effectively treated with the above-mentioned natural substances by physicians. Several of the natural substances used in dentistry are summarized in Table 1.

## 4. Conclusions

Antioxidant-rich natural raw materials and preparations have been used in the prevention of oral diseases including periodontitis and caries. Many scientific studies have confirmed the association between the health of the oral cavity and the composition of the microbiota colonizing it. Based on the available evidence, it can be concluded that natural materials used in dentistry are characterized by a high antimicrobial potential and inhibit the formation of the biofilm and plaque. These substances are mainly used due to their high availability and low cost. However, there is a lack of preclinical and clinical studies evaluating their safety and effectiveness in many cases. The findings presented here confirming the potential of natural substances in the prevention and treatment of oral diseases may pave the way for numerous clinical trials in the future.

## Figures and Tables

**Table 1:** Natural substances used in the prevention and treatment of oral diseases.

Raw Material/Compound	Active Substance with Antioxidant Activity	Disease	Therapeutic Effect	Reference
Allium sativum extract	Allicin, alkaloids, saponins, flavonoids, tannins, steroids	Periodontal and dental caries infections	Antimicrobial properties	[50,51,52,53]
Clove oil	Eugenol	Caries, endodontic treatment	Disinfectant, local anesthetic, promotes healing	[31,55]
Zinc ore	Zinc oxide	Caries, pulp diseases	Bactericidal, bacteriostatic (in combination with eugenol), hygroscopic, promotes remineralization of dentine, receding of initial inflammation of pulp, regenerating, astringent, antioxidant	[31,34,35,55]
Natural rubber isomer	Gutta-percha	Endodontic treatment		[34,55]
Mastic resin	Pinene, resenes, resinic, mastic acid	Caries	Disinfecting	[33,34]
Balsam tree	Myrrh	Periodontal diseases, caries	Anti-inflammatory	[38,58]
Turpentine	Rosin	Caries	Improves plasticity of filling materials	[81]
Oak bark	Tannins, polyphenols, flavonoids	Inflammation, ulcers of the oral cavity	Antioxidant, anti-inflammatory, astringent	[23,35]
Sage leaf	Essential oil containing thujone, cineole, camphor, borneol, pinene, flavonoids, tannins, bitters, organic acids, vitamins A, B_1_, C, and PP	Gingivitis and periodontitis, thrush, streptococcal infections	Antibacterial, anti-inflammatory, antiseptic, astringent, disinfectant, bactericidal	[35,38]
Raspberry leaf	Tannins, polyphenols, vitamin C, fruit acids	Ulcers, inflammation of the oral cavity	Strongly astringent	[33]
Chamomile flower, chamomile oil	Chamazulene, α-bisabolol, spiroether, flavonoid, coumarins, mucilage	Inflammation of the oral cavity and mucosa, periodontitis	Anti-inflammatory, anti-allergic, disinfectant (antibacterial, antifungal), analgesic, alleviating, antiseptic	[22,38]
Peppermint herb, peppermint oil	Menthol		Anesthetic, antibacterial, disinfectant, anti-inflammatory, antibacterial, cooling sensation, analgesic, inhibits bacterial growth, plaque inhibitor	[13,38,82]
Thyme herb	Thymol	Inflammation of the oral cavity	Antibacterial, dehydrating, bactericidal, anti-inflammatory, plaque inhibitor	[13,82]
Eucalyptus oil	Eucalyptus oil (eucalyptol)		Antiseptic, reduces muscle pain and rheumatic complaints, antibacterial, has aromatic smell, disinfectant, anti-inflammatory, plaque inhibitor	[13,83]
Lemon balm leaf	Balm oil, tannins		Antibacterial, antiviral, sedative	[33]
Elecampane rhizome	Essential oil, inulin, phytosterols, mineral salts	Inflammation of the gum and after extraction	Expectorant, diastolic, antiseptic	[33]
Fennel seed		Gum inflammation	Antiseptic	[33]
Chinese cinnamon bark		Gum inflammation	Diastolic, antiseptic, astringent	[46]
Lemon fruit	Essential oils, vitamin C		Cleansing, whitening	[33]
Yarrow herb	Essential oil, flavonoids, tannins, choline, mineral compounds, vitamin C		Disinfecting, healing, anti-inflammatory	[82]
Marshmallow root		Mouth ulcers	Alleviating, soothing, relieving	[33]
Greater burdock root		Mouth ulcers	Antibiotic, purification of blood and other body fluids	[83]
Field horsetail herb	Flavonoids (rutin, quercetin, hesperidin) Horsetail herb: flavonoids, mineral substances, potassium salts, organic acids, saponins, phytosterols, vitamin C, carotenoids, tannins		Anti-inflammatory, similar to vitamin P, antihemorrhagic, regenerative	[84]
Tannin		Increased saliva production		[67]
Aloe vera leaf	Anthraquinones (barbaloin, isobarbaloin, anthranols, aloetic acid)	Inflammatory diseases, gingivitis, periodontitis	Anti-inflammatory, regulating, stimulates immune processes, antibacterial, antiseptic, reduces bleeding, immunomodulatory, antiviral, antifungal	[23,35,44,56]
Sunflower seed oil	Linoleic acid		Hydrating	[35,85]
Laurel oil			Analgesic, bactericidal	[35]
Tea tree oil			Antibacterial, antifungal	[83]
Savory herb extract			Analgesic	[35]
Vitamin E			Strengthens and regenerates epithelium	[35]
Vitamin A, palmitate				[5,35]
White willow bark	Salicylic acid	Acne	Superficial epidermal peeling, cytotoxic, antibacterial	[67,86]
Deep-sea shark liver oil	Complex fats, higher unsaturated fatty acids, alkylglycerols, squalene, vitamins A and D_3_		Infections, autoimmune diseases, periodontitis	[80]
Linseed	α-Linolenic acid	Xerostomia, hypersensitivity to stimuli, e.g., Hunter’s tongue, lichen planus, mucositis	Alleviating effect	[5,63,87,88]
Propolis	Flavonoids, aromatic esters, terpenes	Candidiasis, caries, viral infections, wounds, inflammation of the mucous membranes	Anticaries, bactericidal, bacteriostatic, antiviral, fungicidal	[5,22,57]
Curcumin			Anti-inflammatory, anticancer, antioxidant, scavenges free radicals	[22]
Tormentil rhizome	Catechin tannins	Stomatitis, bleeding, and exudative gingivitis	Astringent, antibacterial, anti-inflammatory	[89]
